# Current Single-cell Proteins as Ingredients: Functions, Challenges, and Regulatory Frameworks

**DOI:** 10.4014/jmb.2606.06024

**Published:** 2026-09-02

**Authors:** Sehyeon Park, Taeyeon Kim, Soyeon Lee, Pil Kim

**Affiliations:** 1 Research Group of Novel Food Ingredients for Alternative Proteins, The Catholic University of Korea, Bucheon, Gyeonggi 14662, Republic of Korea; 2 Department of Biotechnology, The Catholic University of Korea, Bucheon, Gyeonggi 14662, Republic of Korea

**Keywords:** Single-cell protein, Alternative protein, Microbial biomass, Functional ingredients, Regulatory framework

## Abstract

Single-cell proteins (SCPs) broadly comprise microbial biomass or biomass-derived proteinaceous products produced from bacteria, yeasts, filamentous fungi, or microalgae. In the broader field of cell-derived alternative proteins, animal- and plant-cell-derived platforms are also being explored because they can reproduce the structural, compositional, and sensory equality of conventional food materials. In contrast, microbial SCPs are distinguished by diverse cellular structures, metabolites, cell-wall components, and bioactive fractions that may confer functionality beyond nutritional supplementation. This review summarizes recent progress in SCP-related platforms by first contrasting animal- and plant-cell-derived proteins with microbial SCPs, and then focusing on bacterial, yeast, filamentous fungal, and algal SCPs. Recent animal, human, and cell-based studies indicate that microbial SCPs and related microbial biomasses may modulate the gut microbiota, intestinal barrier function, immune and oxidative-stress responses, lipid metabolism, muscle protein synthesis, disease resistance, and product-quality traits. Nevertheless, microbial SCP development remains constrained by microbial-group-specific safety and processing issues, including nucleic acid reduction, toxin and contaminant control, and cell-wall-associated digestibility. Finally, this review summarizes regulatory frameworks for SCPs in the United States and the European Union, where commercialization remains product-specific rather than governed by a harmonized SCP category. Overall, SCPs represent promising but heterogeneous food and feed platforms that require integrated evaluation of nutritional value, functionality, safety, processing, and regulatory compliance.

## Introduction

Conventional protein production is increasingly constrained by environmental and climate-related pressures. Whole food-system accounting indicates that food systems contribute a substantial fraction of total anthropogenic greenhouse gas emissions, with agricultural production and livestock accounting for the dominant share and land-use change imposing additional burdens [1, 2]. These pressures intersect with climate change and land degradation, which directly affect agricultural productivity, soil health, and water availability and quality. Accordingly, there is a growing impetus to expand sustainable protein sources that can meet nutritional requirements within environmental limits [3].

In response, alternative proteins have progressed from niche concepts to a major domain of food-system innovation [4]. Among the leading candidates, insect proteins have received considerable attention, although important barriers remain, including consumer acceptance, production standardization, and allergenicity concerns [5]. In parallel, single-cell proteins (SCPs) produced through high-density microbial cultivation have emerged as a scalable and technologically flexible platform. SCPs are broadly defined as microbial biomass or biomass-derived proteinaceous products produced through microbial cultivation for direct consumption or for use as food and feed ingredients [6]. Animal-cell platforms, commonly discussed as cultured or cultivated meat, aim to reproduce the cellular composition and sensory or nutritional attributes of conventional meat, whereas plant-cell and plant-tissue culture platforms use edible plant cells as biomass, ingredients, or sources of food-relevant compounds. Their main conceptual advantage is potential compositional continuity with familiar animal- or plant-derived foods [7, 8]. However, these platforms remain distinct from microbial SCPs and still face major constraints related to scale-up, culture-medium cost, process efficiency, product validation, and regulatory acceptance. Microbial platforms exhibit considerable metabolic flexibility toward renewable resources and non-edible substrates and can be produced through controlled fermentation processes that enable year-round production with consistent composition [9-12]. Microbial SCPs have already reached commercial food and feed applications, ranging from established products such as fungal mycoprotein to emerging bacterial and gas- or electricity-based protein production systems. Recent techno-economic analyses further indicate that the economic competitiveness of emerging SCP processes may improve with increased production scale, greater process efficiency, and access to low-cost renewable energy and feedstocks [13, 14]. Despite these advantages, substantial challenges remain before SCPs can be commercialized at a scale and cost structure capable of competing with mainstream protein sources. In this context, numerous studies have investigated SCP development, safety, processing, and functionality.

This review summarizes recent studies on the functional properties, major development challenges, and regulatory status of SCPs for food and feed applications. This structure is intended to help readers evaluate SCPs not as a uniform alternative-protein category, but as distinct food and feed platforms that must simultaneously satisfy nutritional, functional, processing, safety, and regulatory requirements. This narrative review was developed through iterative literature searches of PubMed and Google Scholar, supplemented by reference-list screening and searches of publisher and regulatory websites.

## Functional Properties of Cell-Derived Protein Platforms

In this review, the term “platform” refers to the cellular source category from which the protein ingredient or biomass is derived, namely animal, plant, bacterial, yeast, filamentous fungal, or algal cells. Cell-derived protein platforms differ substantially in biological origin and functional positioning. Accordingly, Section 2.1 briefly considers animal- and plant-cell-derived platforms primarily in terms of their structural, compositional, and sensory resemblance to conventional food materials, whereas Section 2.2 focuses on microbial SCPs and related biomass preparations with potential host-response-regulating and postbiotic-like functionality. The microbial SCP literature considered here includes direct human-food studies, animal-feed applications, and preclinical or cell-based studies that provide functional or mechanistic evidence relevant to food and feed development.

Searches combined source-related terms, including single-cell protein, microbial protein, bacterial meal, yeast protein, mycoprotein, Spirulina, *Chlorella*, and *Nannochloropsis*, with outcome-related terms such as food, feed, gut microbiota, intestinal health, immunity, oxidative stress, lipid metabolism, muscle protein synthesis, disease resistance, and product quality. Peer-reviewed studies published primarily between 2020 and 2026 were prioritized. Studies were included when they evaluated SCPs or related cell-derived preparations in human or animal studies or in cell-based models and reported host-response-regulating effects or application-relevant nutritional and cellular outcomes. Studies focused solely on biomass production, process yield, or proximate composition without relevant functional evaluation were generally excluded. Both food- and feed-related studies were considered. Earlier studies were retained when they provided foundational evidence or established functional benchmarks. The animal- and plant-cell studies were selected as representative, rather than exhaustive, examples that directly evaluated structural, compositional, or sensory resemblance to conventional food materials.

### Animal- and Plant-Cell-Derived Proteins

Animal- and plant-cell-derived protein platforms differ from microbial SCPs in that their main functional advantage lies in their continuity with conventional food materials. In animal-cell platforms, cultured meat studies have attempted to reproduce the structural and sensory basis of conventional meat by controlling the differentiation and assembly of muscle and adipose cells. For example, bovine myoblasts and adipose-derived mesenchymal stem cells were differentiated on gelatin/alginate scaffolds and assembled into cultured meat with sensory profiles similar to conventional beef, whereas engineered bovine adipose tissue was integrated with muscle tissue to mimic inter- and intramuscular fat structures, key determinants of meat flavor, juiciness, tenderness, texture, and nutritional quality [15, 16].

Plant-cell platforms follow a similar logic of food-material equivalence. Bioreactor-grown coffee cells have been dried and roasted to generate coffee-like aroma and flavor, with chemical and sensory analyses used to compare the resulting brews with conventional coffee [17]. In other plant cell cultures, such as rowan and arctic bramble, processing and formulation improved mouthfeel, texture, and flavor while providing substantial protein and dietary fiber contents [18]. Therefore, animal- and plant-cell platforms are best positioned as cellular agriculture systems that seek structural, compositional, and sensory resemblance to established foods (Table 1).

### Microbial SCPs

Unlike animal- and plant-cell-derived protein platforms, whose major advantage lies in their compositional proximity to conventional food sources, microbial SCPs are functionally distinguished by their diverse cellular structures, metabolites, cell-wall components, and bioactive fractions. Accordingly, microbial SCPs may act not only as protein ingredients but also as biologically active ingredients capable of modulating host responses, including the gut microbiota, intestinal barrier function, immune and oxidative-stress responses, lipid or muscle metabolism, and disease resistance. These properties overlap conceptually with postbiotic-like functionality and provide the basis for evaluating bacterial, yeast, filamentous fungal, and algal SCPs as functionally distinct microbial ingredients [19].

**Bacterial SCPs.** Bacterial SCPs are produced from metabolically diverse microorganisms, including methanotrophs, gas-fermenting bacteria, and amino acid-producing bacteria. Their functional relevance is increasingly evaluated in terms of host-response regulation and application-enabling nutritional or cellular functions [20]. Therefore, representative bacterial SCP studies can be considered according to whether the evaluated SCP preparation modulates intestinal integrity, the gut microbiota, immune or oxidative-stress responses, lipid or muscle metabolism, or cellular functionality relevant to food and feed applications (Table 2).

Methanotroph-derived bacterial meal has been investigated mainly as a protein-replacing ingredient in aquafeed. Among these studies, *Methylococcus capsulatus*-based SCP is notable because selected evidence suggests functional effects beyond growth performance. In spotted sea bass, dietary *M. capsulatus* biomass attenuated soybean-meal-induced enteritis and was associated with improved intestinal immune responses and gut microbiota modulation [21]. Gas-fermentation-derived *Clostridium autoethanogenum* protein has also been evaluated as an alternative bacterial protein source. In broilers, dietary *C. autoethanogenum* protein affected growth performance, lipid metabolism, the gut microbiota, short-chain fatty acids, bile acid metabolism, and beneficial bacterial populations [22]. Another broiler study further linked this bacterial protein to improved growth and muscle protein synthesis through activation of the mTOR signaling pathway [23]. These findings indicate that bacterial SCPs may influence host physiology through gut- and tissue-level responses rather than functioning solely as crude protein substitutes.

*Corynebacterium glutamicum* is a Gram-positive, non-pathogenic industrial bacterium widely used for amino acid fermentation. Pilot-scale *C. glutamicum* SCP showed high protein content, low fat content, measurable heme iron, and a Digestible Indispensable Amino Acid Score (DIAAS) of 102, indicating high protein quality compared with several conventional and alternative protein sources [24]. In gilthead sea bream, dietary bacterial SCP derived from *C. glutamicum* supported protein replacement without impairing growth, apparent protein digestibility, plasma biochemical parameters, intestinal morphology, or the gut microbiota [25]. Terrestrial animal studies further support the functional relevance of *C. glutamicum*-derived cell mass. In nursery pigs, lysed *C. glutamicum* cell mass was associated with intestinal health and growth-related effects [26], whereas amino acid fermentation-derived products containing *C. glutamicum* cell mass improved nutrient utilization, jejunal immune and oxidative-stress status, and growth or health-related parameters [27, 28]. These studies suggest that *C. glutamicum*-derived bacterial materials may serve not only as nutrient sources but also as host-response-regulating ingredients.

Heme-enriched *Corynebacterium* materials provide another example of bacterial biomass functionality. Single-cell hemoprotein, or heme-SCP, showed prebiotic potential by supporting a healthier gut microbiota profile in small pet dogs [29]. In high-fat diet-induced obese mice, heme-SCP altered adipose tissue distribution and reshaped the gut microbiota [30]. Dietary heme-enriched *Corynebacterium* extract was also reported to improve lipid- and gut-health-related parameters through gut microbiota reshaping [31]. Beyond these animal studies, heme-enriched *Corynebacterium* extracts have been applied in cell-culture systems for cultivated meat. These studies indicated that bacterial heme extracts could support heme adaptation in porcine and bovine cells under reduced-serum or serum-free culture conditions, with redox- and detoxification-related cellular responses implicated in heme tolerance [32, 33]. Although these studies are not conventional SCP feeding trials, they are relevant to bacterial SCP development because they demonstrate application-enabling cellular functionality of bacterial biomass-derived components.

Recent postbiotic-oriented studies also extend the functional interpretation of *C. glutamicum*-derived materials. A lysate supernatant of *C. glutamicum* repaired inflammatory barrier dysfunction in intestinal epithelial cells and attenuated colitis-related responses in mice, with effects involving inflammatory cytokine regulation, tight-junction and mucin-related markers, oxidative-stress reduction, cell-cycle recovery, and FOXO/L-arginine-associated mechanisms [34]. Similarly, pasteurized *Akkermansia muciniphila* is not a bulk protein-source SCP but provides a useful benchmark for bacterial biomass functionality because its reported effects are mediated primarily through host-response regulation rather than nutrient equivalence. Preclinical and human studies have linked pasteurized *A. muciniphila* to improvements in metabolic, inflammatory, and gut-barrier-related parameters, although the human evidence remains exploratory and does not indicate broad restructuring of the gut microbiome [35, 36]. These examples support the broader concept that processed or non-viable bacterial biomass preparations can act as biologically active ingredients.

Overall, bacterial SCPs show functional potential in gut-barrier regulation, microbiota modulation, immune and oxidative-stress responses, lipid or muscle metabolism, and cell-culture-related applications.

**Yeast SCPs.** Yeast-derived SCPs combine established food or feed uses with microbe-specific functional properties. In addition to supplying protein, amino acids, B-complex vitamins, minerals, and cell-wall polysaccharides, yeast SCPs may regulate host responses through effects on gut microbiota, intestinal immune activity, oxidative status, epithelial function, and disease resistance [17, 37]. Therefore, representative yeast-SCP studies should be interpreted not only as protein-replacement trials, but also as evidence for host-response regulation and applicable nutritional functionality (Table 3).

*Cyberlindnera jadinii*, commonly referred to as Torula yeast, is one of the most extensively evaluated yeast SCPs. In zebrafish, dietary *C. jadinii* modulated immunoregulatory pathways in the intestinal proteome and improved immunity and survival against *Aeromonas* infection, indicating functional activity beyond basic protein substitution [38, 39]. Terrestrial animal studies further support this interpretation. In post-weaning piglets, *C. jadinii* used as a protein source affected the intestinal microbiota, butyrate levels, immune response, and gut microbial composition [40, 41]. A more recent piglet study also reported that a diet containing *C. jadinii* produced benefits comparable to those of a formic acid and probiotic-supplemented diet, suggesting that this yeast biomass may provide functional effects relevant to post-weaning intestinal adaptation [38]. In companion animals with chronically poor stool quality, Torula yeast affected apparent total tract digestibility, inflammatory markers, and fecal microbiota dysbiosis, further supporting its relevance as a gut-health-oriented yeast ingredient [43].

*Saccharomyces cerevisiae*-derived ingredients have also been evaluated for functional effects, although their effects depend strongly on product form, inclusion level, and target species. In channel catfish, partial replacement of dietary fishmeal with *S. cerevisiae* culture improved growth performance, immunity, disease resistance, and the composition and functional potential of the intestinal microbiota [44]. This study is more informative than simple growth-based replacement trials because it links yeast culture to immune competence, pathogen resistance, and microbiota-associated host responses. Thus, *S. cerevisiae*-derived SCPs or yeast cultures may function as both alternative protein ingredients and immunonutritional feed components when appropriately formulated.

Other non-*Saccharomyces* yeasts provide additional examples of functional diversity. *Yarrowia lipolytica* has been evaluated both as a protein-replacing ingredient and as a functional yeast supplement. In Pacific white shrimp and largemouth bass, dietary *Y. lipolytica* preparations, including autolyzed biomass in the largemouth bass study, maintained or improved growth-related outcomes at appropriate inclusion levels while affecting nutrient digestibility, antioxidant capacity, digestive enzyme activity, serum lipid-related metabolites, intestinal morphology, gut microbiota, and transcriptomic pathways associated with growth, metabolism, and immunity. However, excessive inclusion impaired feed utilization, growth, antioxidant activity, or intestinal morphology, indicating that its functional value is dose-dependent [45, 46]. In Nile tilapia and rainbow trout, *Y. lipolytica* functioned more clearly as an immune-oriented supplement: fermented biomass stimulated innate immune responses in Nile tilapia, whereas whole yeast supplementation upregulated intestinal immune-related genes in rainbow trout [47, 48].

*Debaryomyces hansenii* also illustrates the potential of yeast SCPs to modulate host–microbiota interactions. In gilthead sea bream fed a low-fishmeal diet, low-level *D. hansenii* supplementation improved growth and feed conversion while modulating the gut microbiota, mucin-related intestinal features, and intestinal transcriptomic pathways associated with metabolic, antioxidant, immune, and host–microbiota interactions [49]. This suggests that some yeast SCPs may contribute to intestinal adaptation under reduced fishmeal conditions, not only by supplying nutrients but also by influencing epithelial and microbial responses.

Overall, yeast SCPs show functional potential through modulation of the gut microbiota, intestinal immune responses, antioxidant status, disease resistance, and nutrient utilization.

**Filamentous fungal SCPs.** Filamentous fungal SCPs differ from bacterial and yeast SCPs in both biomass structure and application context. Their mycelial morphology, cell-wall composition, protein content, and fibrous matrix can contribute not only to protein supply but also to texture, satiety, gut fermentation, and host metabolic responses [46, 51]. Therefore, representative filamentous fungal SCP studies are best interpreted according to both applicable nutritional functionality and regulation of host responses (Table 3).

*Fusarium venenatum*-derived mycoprotein, commercially known as Quorn, is the most extensively studied food-grade filamentous fungal SCP. Human studies indicate that mycoprotein provides substantial postprandial amino acid availability among non-animal protein sources, although aging may delay, rather than reduce, the overall plasma amino acid response [52]. Mycoprotein also supports muscle protein metabolism. Acute mycoprotein ingestion, mycoprotein-rich diets, and mycoprotein-derived protein concentrates have been reported to stimulate resting, daily, or post-exercise myofibrillar protein synthesis and to support resistance-training-induced improvements in lean mass, thigh muscle volume, muscle fiber size, and strength [53-58]. These findings support the role of *F. venenatum* mycoprotein as an applicable nutritional platform for muscle protein remodeling, rather than merely as a meat-replacement ingredient.

Mycoprotein has also been evaluated for cardiometabolic and gut-health-related effects. Short-term substitution of meat or fish, including red and processed meat with mycoprotein reduced total cholesterol, low-density lipoprotein cholesterol, or other cholesterol-containing lipoprotein markers in several human intervention studies, although effects on insulin sensitivity, free-living glycemic control, blood pressure, and appetite-related outcomes were less consistent [59-61]. Acute meal studies further showed that mycoprotein can alter postprandial glucose kinetics and reduce postprandial glucose responses in adults with type 2 diabetes, but these changes were not always accompanied by lower insulin responses or appetite-related effects [62, 63]. With respect to gut health, replacing red and processed meat with mycoprotein reduced fecal genotoxicity and nitroso-compound excretion while increasing short-chain fatty acid excretion and beneficial taxa, including Lactobacilli, Roseburia, and Akkermansia [64]. These findings indicate that *F. venenatum* mycoprotein may regulate host responses through lipid metabolism, glycemic handling, microbial fermentation, and gut microbial composition.

*Paecilomyces variotii*-derived SCP, known as PEKILO, has recently re-emerged as a filamentous fungal SCP for food and feed applications. In Atlantic salmon, dietary *P. variotii* maintained growth performance and feed intake while improving feed conversion, nutrient-retention efficiency, gut health, immune-related responses, and gut microbiota modulation [65, 66]. However, increasing inclusion reduced the apparent digestibility of crude protein, gross energy, and amino acids in one study, indicating that its nutritional value depends strongly on inclusion level and diet formulation [65]. In adult dogs, *P. variotii* SCP showed acceptable crude-protein digestibility and did not impair palatability or fecal score, although higher inclusion reduced some diet-level digestibility coefficients. It also modulated fecal short-chain fatty acids and the gut microbiota, including increases in beneficial bacterial genera and reductions in potentially undesirable taxa [67]. These studies suggest that PEKILO may provide both protein functionality and gut-response modulation, but its application requires careful control of digestibility, dose, and matrix effects.

Overall, filamentous fungal SCPs provide specific functionality related to amino acid availability, muscle protein synthesis, lipid and glucose metabolism, gut fermentation, digestibility, and microbiota modulation.

**Algal SCPs.** Algal SCPs, including cyanobacterial and microalgal SCPs, are functionally distinct from bacterial, yeast, and filamentous fungal SCPs because their value is not limited to protein supply. In addition to proteins and essential amino acids, algal SCPs may provide pigments, carotenoids [68], polyunsaturated fatty acids, vitamins, minerals, polysaccharides, and antioxidant compounds [69]. Therefore, representative algal SCP studies should be interpreted according to both applicable nutritional functionality and regulation of host responses, particularly antioxidant status, immune function, the gut microbiota, intestinal integrity, disease resistance, lipid metabolism, and product quality (Table 4).

*Arthrospira platensis*, commonly referred to as Spirulina, is one of the most widely studied algal SCPs. In Pacific white shrimp, dietary Spirulina improved growth-related traits while enhancing antioxidant activity, tissue histology, body composition, and resistance to *Vibrio parahaemolyticus*, indicating functional relevance beyond simple protein supplementation [70]. In zebrafish, Spirulina affected growth, reproductive performance, and metabolic responses through nutrigenomic modulation, suggesting that its effects may involve host metabolic regulation rather than only nutrient replacement [71]. Terrestrial animal studies further support its functional potential. In broilers, Spirulina supplementation modulated molecular, immunohistopathological, and gut microbiota-related responses [72]. Under aflatoxin B1 challenge, Spirulina also improved immune and antioxidant status and alleviated pathological damage, indicating potential stress-protective functionality [73]. These findings support the interpretation of Spirulina as an algal biomass with antioxidant, immunomodulatory, microbiota-regulating, and disease- or toxin-resilience-related effects.

*Chlorella vulgaris* represents another major algal SCP platform, with functional evidence in both aquatic and terrestrial models. In aquatic animals, dietary *C. vulgaris* has been associated with modulation of growth, antioxidant capacity, immune responses, intestinal function, the gut microbiota, stress resistance, disease resistance, and muscle- or product-quality-related traits [74-77]. These outcomes suggest that *C. vulgaris* may support host-response regulation through antioxidant and immune pathways, while also contributing to nutrient utilization and tissue quality. In weaned piglets, *C. vulgaris* supplementation reduced diarrhea and affected lipid- and inflammation-related parameters, including increased high-density lipoprotein cholesterol and reduced ileal TNF-α expression, together with changes in colonic microbiota and metabolites [78]. This terrestrial evidence is particularly useful because it links algal biomass intake to intestinal health, inflammatory status, and microbial-metabolic responses rather than only growth performance.

*Nannochloropsis* spp. provide a distinct algal SCP model because of their lipid fraction, eicosapentaenoic acid content, carotenoids, and potential contribution to product-quality improvement. In Atlantic salmon, dietary *Nannochloropsis oceanica* allowed partial replacement of conventional marine ingredients while maintaining EPA and DHA deposition, although lipid digestibility was reduced, indicating both nutritional value and formulation-dependent limitations [79]. In broilers, replacement of soybean protein with *N. oceanica* increased meat n-3 polyunsaturated fatty acids and carotenoids despite some reduction in growth performance, suggesting that its value may lie partly in product enrichment rather than maximal growth promotion [80]. In rats, *N. oceanica* supplementation increased lean mass and reduced fat mass, although broader metabolic-syndrome-related outcomes were not uniformly improved [81]. In Nile tilapia, *Nannochloropsis oculata* improved growth, feed utilization, omega-3-related body composition, antioxidant response, digestive capacity, intestinal immunity, intestinal integrity, and resistance to *Aeromonas* infection in a dose-dependent manner [82-84]. These findings indicate that *Nannochloropsis*-derived SCPs may provide both applicable nutritional functionality and host-response regulation, but their effects depend strongly on dose, digestibility, and the balance between protein and lipid-associated bioactive components.

Overall, algal SCPs show functional potential through antioxidant activity, immune modulation, intestinal protection, disease or stress resistance, lipid metabolism, and product-quality improvement.

### Cross-Platform Interpretation of Functional Properties

The studies summarized in Section 2 indicate that the functional value of cell-derived protein ingredients cannot be inferred from crude protein content alone. Animal- and plant-cell-derived platforms are primarily characterized here by their structural, compositional, and sensory resemblance to conventional food materials, whereas microbial SCPs may provide additional functionality associated with their cellular structures, metabolites, cell-wall components, pigments, lipids, polysaccharides, and other bioactive fractions. However, the functional evidence for microbial SCPs remains heterogeneous. The cited studies differ substantially in organism and strain, production substrate, processing and preparation form, inclusion level, basal diet, host model, and measured endpoints. Quantitative comparisons of protein quality, digestibility, production efficiency, cost, environmental impact, and commercial maturity across SCP categories are also limited because these parameters are inconsistently reported and are often assessed using non-equivalent metrics, analytical methods, production scales, and system boundaries. These differences limit direct comparison among studies and preclude broad generalization of individual functional effects across bacterial, yeast, filamentous fungal, and algal SCPs.

The strength of evidence also differs across study types and microbial sources. Human evidence is currently most substantial for selected filamentous fungal SCPs, particularly *Fusarium venenatum* mycoprotein, whereas much of the evidence for bacterial, yeast, and algal SCPs is derived from animal or cell-based models. Several reported functional effects, particularly gut microbiota modulation, have been observed predominantly in animal studies, and independent replication across comparable experimental conditions remains limited. Mechanistic support is also variable: some studies provide molecular, biochemical, transcriptomic, or pathway-level evidence, whereas others demonstrate associations between SCP intake and physiological or microbial outcomes without establishing causality. Accordingly, current evidence does not support robust quantitative ranking of functional effects across SCP categories because study designs, host species, doses, preparation forms, and endpoints are insufficiently standardized for direct numerical comparison. Future studies should use standardized preparations, appropriate nutritional controls, dose-response designs, and comparable endpoints to determine whether the reported effects are reproducible and relevant under practical food or feed conditions.

## Challenges in Microbial SCP Development

Although each microbial platform has distinct compositional and technological characteristics, SCPs share several features that distinguish them from animal proteins and many other alternative proteins, including elevated nucleic acid contents, microbial-group-specific toxin or contaminant concerns, and microbe-derived cell-wall structures. These factors must be carefully considered when SCPs are applied as major protein components in food or feed. Fig. 1 provides a schematic overview of the major SCP-specific challenges and representative recent strategies used to address them across microbial platforms.

### Nucleic Acid Reduction

Microbial SCPs commonly contain relatively high levels of nucleic acids, partly because microorganisms have small cell sizes and high nucleic acid-to-biomass ratios compared with plant or animal cells. Because dietary nucleic acids are degraded to purine bases and ultimately contribute to uric acid production in humans, excessive intake of nucleic-acid-rich microbial biomass may increase serum or urinary uric acid levels, thereby increasing the risk of disorders such as gout and kidney stones. Therefore, nucleic acid reduction or intake control is generally required for food applications [20].

Bacterial biomass generally contains relatively high nucleic acid levels compared with some other microbial SCP platforms, with reported values of up to 16%. Recent studies have attempted to mitigate this issue through upstream substrate selection and process optimization. With respect to nucleic-acid-related safety, recent studies on *Cupriavidus necator* suggest that biomass composition can be partly controlled by cultivation parameters and substrate selection. Ismail *et al*. showed that temperature and pH significantly affected the composition of *C. necator* SCP, including its protein and nucleic acid fractions [85]. Subsequently, they reported that cultivation on formic acid with ammonium sulfate produced biomass with high protein content and the lowest nucleic acid and polyhydroxyalkanoate contents among the tested conditions [86].

Yeast and filamentous fungal SCPs generally have lower nucleic acid contents than rapidly growing bacterial biomass, but they can still contain considerable levels, with reported values of up to 12% [20]. Classical studies established heat- and acid-based methods for reducing nucleic acids in yeast [87, 88]. However, high-temperature acidic conditions can also cause partial or complete degradation or chemical conversion of labile amino acids [89, 90]. Recent studies have illustrated several nucleic-acid-reduction approaches, including heat activation of endogenous RNases, alkaline treatment, and salt- or buffer-assisted extraction. In commercial *Fusarium venenatum* mycoprotein production, RNA reduction has been incorporated into standard downstream processing. RNA content is reduced from approximately 10% to <2% of dry weight by heating above 68 °C, which activates endogenous RNases. This indicates that endogenous RNase-mediated RNA reduction is already an established practical solution in mycoprotein processing [91]. Recent yeast-SCP studies have also examined chemical treatments. In palmyrah-toddy mixed-culture SCP produced from fruit peel wastes, dried biomass was treated with 1 N NaOH and heated at 100°C for 10 min; however, this treatment caused significant reductions in dry biomass and crude protein content, indicating a trade-off between nucleic acid reduction and product yield [92]. In *Saccharomyces cerevisiae* SCP produced from banana peel hydrolysate, NaCl, NaOH, and Gly-NaOH buffer treatments reduced spectrophotometrically measured nucleic acid concentrations, whereas Gly-NaOH and NaCl were suggested to be more favorable than NaOH because they caused less protein loss [93].

Microalgae have relatively low nucleic acid contents among microbial groups, with reported values generally up to approximately 6%; nevertheless, nucleic acid intake remains relevant when algal biomass is consumed daily as a bulk SCP ingredient [20]. Thus, recent microalgal SCP studies have mainly focused on recovering protein-enriched fractions rather than directly reducing nucleic acids, which can reduce the relative proportion of non-protein cellular components. *Nannochloropsis* biomass was processed by ultrafiltration/diafiltration to recover a soluble protein-enriched fraction with reduced lipid and chlorophyll contents [94]. In *Auxenochlorella protothecoides* biomass, pulsed electric field or ultrasonication followed by incubation improved protein extraction [95]. In a subsequent study, microfiltration was used to recover soluble protein from disrupted *A. protothecoides* biomass, showing that mild pulsed electric field-based disruption reduced membrane fouling compared with severe homogenization [96]. Wet *A. protothecoides* biomass also showed markedly improved protein recovery when high-pressure homogenization was combined with mild alkaline solubilization, whereas alkaline treatment alone was insufficient [97].

### Toxin and Contaminant Control

For Gram-negative bacterial SCPs, lipopolysaccharide (LPS), an endotoxin component of the outer membrane, represents a specific safety concern for food applications [98]. Accordingly, food-oriented bacterial SCP research often emphasizes Gram-positive bacteria or endotoxin-free production approaches, together with toxicological and compositional evaluation. However, direct studies on LPS mitigation in bacterial biomass remain relatively limited. Recent studies have mainly addressed this issue indirectly through safety, tolerance, and inclusion-level evaluations rather than through direct LPS removal from biomass. For example, when SCP derived from the Gram-negative bacterium *Methylococcus capsulatus* is applied in animal feeding studies, safety is generally assessed by evaluating immune- and inflammation-related responses [21, 99, 100].

Although yeast and fungal SCPs avoid the LPS burden associated with Gram-negative bacterial SCPs, some fungal taxa can produce mycotoxins or allergenic proteins. Wild-type strains of *Aspergillus oryzae* have the potential to produce several mycotoxins, including aflatoxins, cyclopiazonic acid, and 3-nitropropionic acid (3-NPA). A recently developed strain lineage for alternative protein production was reported to lack mycotoxin production capacity due to large-scale deletions, including deletion of the 3-NPA biosynthetic genes *npaA* and *npaB* [101]. Thus, genetic exclusion of toxin biosynthesis is technically feasible, but direct SCP-focused evidence remains limited. Therefore, current fungal SCP safety assessment still relies mainly on safe strain selection, genome-level screening, and chemical confirmation of mycotoxin absence. For example, Fusarium-based mycoprotein is the most established mycoprotein platform, but safety must be evaluated at the strain and process levels rather than inferred from the genus alone [102, 103]. In *F. venenatum*, recent studies have included nutritional composition analysis and mycotoxin quantification, showing that detected fumonisin B1 levels were below established regulatory limits, whereas zearalenone and deoxynivalenol were not detected [104].

Cyanobacteria and some microalgae can produce toxic secondary metabolites. For algal SCPs, however, toxin-related concerns generally arise from contamination rather than from toxin production by the intended production strain. Potential hazards include cyanotoxins produced by contaminating cyanobacteria, toxic metabolites from certain algal taxa, heavy metals, and microbiological contaminants introduced during cultivation, harvesting, drying, or storage [105]. For example, retail Spirulina supplements and Spirulina-containing foods have been shown to contain diverse microbial populations and cyanotoxins [106], whereas other commercial microalgal supplements have been reported to contain microcystin-LR, anatoxin-a, microbial contaminants, and elevated levels of some toxic metals [107]. In addition, multi-class cyanotoxin screening of algal dietary supplements marketed in North America has shown that some products contain microcystins or related cyanotoxin markers [108]. Therefore, recent studies on algal food safety emphasize the need for contamination control across the full production chain, including strain authentication, controlled cultivation, water and nutrient-source management, hygienic harvesting, standardized drying, and routine testing for cyanotoxins, toxic metals, and microbiological contaminants [105, 109].

### Cell-Wall-Associated Digestibility

In Gram-positive bacterial SCPs, the thick cell envelope, including peptidoglycan and associated cell-wall polymers, may influence protein digestibility by limiting digestive enzyme access to proteins. Feeding studies using *C. glutamicum* SCP suggest that insufficient cell-wall lysis may reduce protein utilization in some target species, such as flathead grey mullet [110]. This indicates that the digestibility of Gram-positive bacterial SCPs may depend strongly on whether the cell envelope is sufficiently disrupted during processing. In addition, *Nocardioides nitrophenolicus* biomass showed markedly lower protein digestibility than yeast and fungal samples, further suggesting that Gram-positive bacterial cell-wall structure can contribute substantially to differences in protein utilization [111]. Post-processing strategies may improve the accessibility of digestive enzymes to intracellular proteins. Lysed *C. glutamicum* cell mass has been evaluated as a protein supplement in nursery pigs, and scanning electron microscopy (SEM) analysis confirmed increased cell lysis after homogenization [26]. However, intact and lysed cell masses were not directly compared as dietary treatments; mechanical disruption should currently be presented as a plausible approach rather than a fully established solution for improving the digestibility of Gram-positive bacterial SCPs.

Yeast cell walls are composed mainly of β-glucans, mannans, and other polysaccharides, whereas filamentous fungal cell walls contain β-glucans, chitin, chitosan-like polymers, and other structural carbohydrates [112]. These components can provide dietary fiber-like benefits, but they can also reduce protein bioaccessibility by limiting digestive enzyme access [113]. In yeast biomass, autolysis increased protein and amino acid digestibility in Atlantic salmon in a species-dependent manner, indicating that downstream processing influences nutrient availability [114]. For filamentous fungal SCP, direct cell-wall engineering has recently been investigated. Deletion of a chitin synthase gene in *F. venenatum* reduced chitin content from 8.56% to 6.29%, increased protein content from 35.30% to 54.12%, shortened gastric half-emptying time, and improved protein digestibility and the essential amino acid index [115]. Together, these studies indicate that the digestibility limitations of yeast and filamentous fungal SCPs can be partly addressed through post-processing and targeted modification of cell-wall composition.

Algal cell-wall structures range from glycoprotein-rich coverings and cellulose-based walls to algaenan-containing resistant layers, polysaccharide-rich mucilage, and silica-associated structures. For example, many green algae contain cellulose, hemicellulose-like polysaccharides, pectic compounds, and glycoproteins, whereas some *Chlorella*-related algae possess resistant algaenan-rich outer layers. *Nannochloropsis* has a thick bilayered wall containing cellulose and algaenan-like components, *Porphyridium* is surrounded by polysaccharide-rich mucilage, and diatoms such as *Phaeodactylum* possess silica-based frustules [116]. These cell-wall components can act as dietary fiber-like materials, but they may also reduce protein bioaccessibility by limiting digestive enzyme access to intracellular proteins [117]. Enzymatic treatment of *Chlorella vulgaris* cell walls with chitinase, rhamnohydrolase, and galactanase increased protein bioaccessibility from 49.2% to 58.7% [118]. Mechanically disrupted green microalgal biomass exhibited higher apparent, true, and *in vitro* protein digestibility than undisrupted biomass in a rat feeding study [119]. High-pressure homogenization and bead milling of *C. vulgaris* biomass improved protein solubility and increased *in vitro* protein digestibility from approximately 65% to more than 80% [120]. Bead-milled *Chloroidium saccharophilum* biomass showed a marked increase in total protein digestibility from 62.2% to 99.7%, with improved amino acid digestibility and *in vitro* DIAAS values [121]. Together, these studies demonstrate that microalgal protein digestibility can be improved by mechanical or enzymatic disruption of the cell wall.

## International Regulatory Frameworks for SCP Use in the Food and Feed Sectors

At present, SCPs are not regulated internationally under a single harmonized “single-cell protein” category. Instead, microbial biomass products are evaluated on a case-by-case basis according to the producing organism, production process, substrate, intended use, compositional specifications, and expected exposure. This results in a fragmented regulatory landscape in which safety must be demonstrated for each product and application. This section summarizes the current regulatory status of SCPs under the Food and Drug Administration (FDA) in the United States and the European Food Safety Authority (EFSA) in the European Union (Fig. 2).

### United States/FDA

In the United States, SCPs are regulated through existing food or feed pathways rather than through an SCP-specific approval system. For human food, SCPs may be evaluated under the generally recognized as safe (GRAS) framework. Under this pathway, a substance that is generally recognized as safe under its intended conditions of use is not subject to FDA premarket approval as a food additive. However, this does not constitute formal FDA approval; rather, FDA may review a GRAS notice and issue a “no questions” response to the notifier’s GRAS conclusion [122]. In practice, this framework has allowed some microbial protein ingredients to receive FDA “no questions” responses. For example, fungal protein from *Fusarium* sp. mycelia received a GRAS “no questions” letter in 2022 for use as a food ingredient, excluding meat, poultry, and infant formula, at levels up to 90% of the final product (GRN No. 945). For animal feed, the FDA Center for Veterinary Medicine maintains an animal food GRAS notice inventory. This inventory includes several microbial-derived feed ingredients, including dried *Methylobacterium extorquens* biomass for aquaculture crustaceans and dried *M. capsulatus* product for salmonid diets. Notably, the FDA issued a “no questions” response for dried *M. capsulatus* product intended as a protein source in salmonid diets at up to 18% of the diet (AGRN No. 60).

Thus, FDA evaluation is relatively flexible but product- and use-specific rather than platform-wide. A microbial species with a history of safe use does not automatically render all biomass products derived from that species safe; safety depends on the strain, substrate, residual contaminants, manufacturing controls, composition, exposure level, and target population or animal species. Therefore, microbial SCPs require evidence supporting their safety under the intended conditions of use [123].

### European Union/EFSA

In the European Union, SCPs intended for human food are commonly evaluated under the Novel Food Regulation by the NDA Panel if they were not consumed to a significant degree in the EU before May 15, 1997. EFSA’s updated novel food guidance requires information on identity, production process, compositional data, specifications, proposed uses, intake, nutritional information, toxicology, and allergenicity [124]. This is especially relevant to SCPs because the same microbial species can produce different products depending on the strain, substrate, and downstream processing. The EU status of microbial foods is therefore heterogeneous. Some products may be considered non-novel because of their history of use, whereas others require novel food authorization. For example, the European Commission consultation list classified mycoprotein derived from *F. venenatum* strain A 3/5 as ‘Not Novel Food’ in 2024, whereas mycoprotein from *F. venenatum* strain NRRL 26228 was classified as a ‘Novel food’. For feed-related applications, EFSA evaluates safety and efficacy before authorization when a product is treated as a feed additive, and the FEEDAP Panel assesses safety for target animals, consumers of animal products, users, and the environment [125-129]. Thus, EFSA functions as the scientific risk-assessment body, whereas market authorization is handled through the European Commission and broader EU regulatory procedures.

For microorganisms used in the food or feed chain, EFSA also applies the Qualified Presumption of Safety (QPS) approach. QPS is a preassessment system for biological agents intentionally added to food or feed, but it is not an unconditional approval; microorganisms may receive QPS status only with qualifications, such as absence of acquired antimicrobial resistance or toxigenic potential [130]. EFSA has also emphasized whole-genome sequencing (WGS)-based characterization of microorganisms intentionally used in the food chain, which is directly relevant to SCP development because genome analysis can support strain identification and screening for antimicrobial-resistance genes, virulence factors, and toxin-biosynthetic potential [131-133].

Overall, the current regulatory status of SCPs is advanced but not harmonized. Commercialization is possible in major markets, but approval depends on product-specific evidence for strain identity, production process, composition, contaminants, toxic metabolites, nucleic acids, allergenicity, digestibility, and intended intake level.

## Conclusion

SCPs and related cell-derived protein platforms should not be regarded as a uniform alternative-protein category. Animal- and plant-cell-derived platforms are primarily distinguished by their potential structural, compositional, and sensory resemblance to conventional food materials, whereas microbial SCPs are characterized by properties of microbial biomass that differ among cellular sources. Bacterial, yeast, filamentous fungal, and algal SCPs differ markedly in cellular structure, metabolites, cell-wall composition, bioactive fractions, digestibility, and safety concerns. Accordingly, their value cannot be inferred from crude protein content alone.

Recent studies indicate that microbial SCPs may provide functional effects beyond protein supplementation, including modulation of the gut microbiota, intestinal barrier function, immune and oxidative-stress responses, lipid metabolism, muscle protein synthesis, disease resistance, and product quality. However, because these effects depend on microbial source, strain, processing and preparation form, dose, and host, they should not be generalized across SCPs. Therefore, functional claims require careful interpretation and should be supported by standardized preparations, nutritional controls, dose-response designs, and comparable biological endpoints.

Successful SCP development also requires resolution of core technical and safety issues. Nucleic acid reduction, toxin and contaminant control, and cell-wall-associated digestibility remain central challenges, although their relative importance differs among bacterial, yeast, fungal, and algal platforms. Regulatory pathways in the United States and the European Union further reinforce the need for product-specific evidence, because SCPs are evaluated according to strain identity, production process, composition, intended use, exposure, and safety rather than as a harmonized category. Overall, SCPs are promising food and feed ingredients, but their practical application depends on integrated evaluation of nutritional quality, biological functionality, processing feasibility, safety, and regulatory compliance.

## Figures and Tables

**Fig. 1:**
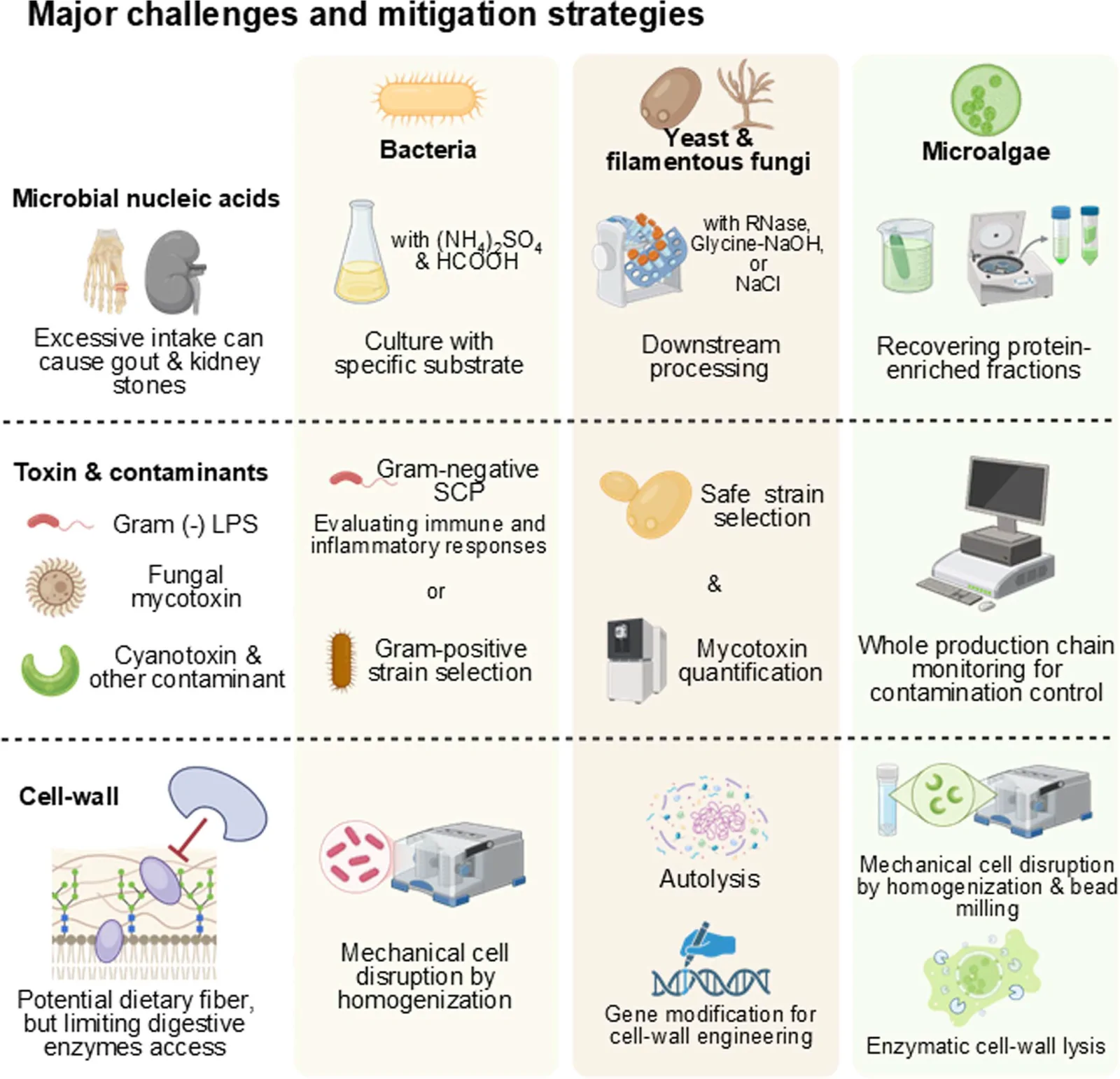
Major challenges in SCP development and representative mitigation strategies across microbial platforms. Created in BioRender. Park, S. (2026) https://BioRender.com/mwq86l0.

**Fig. 2:**
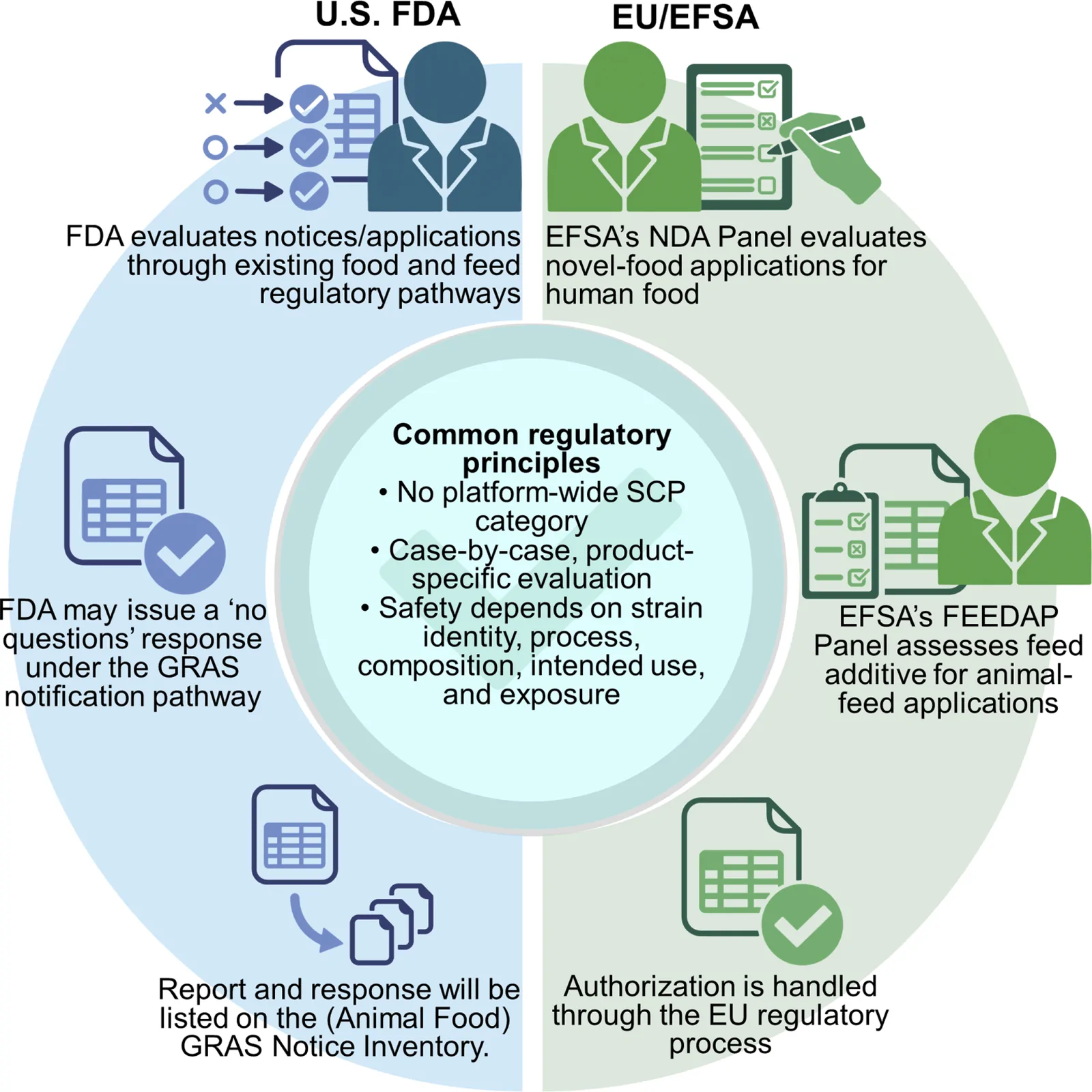
Regulatory frameworks for the use of SCPs in the food and feed sectors. Created in BioRender. Park, S. (2026) https://BioRender.com/n5punxf.

**Table 1:** Food-material equivalence of animal- and plant-cell-derived protein.

Single cell type	Target model [Ref.]	Functional outcomes
Bovine myoblasts and bovine adipose-derived mesenchymal stem cells	Cultured meat construct [13]	• Reproduction of beef-like sensory profiles • Regulation of muscle and adipose cell differentiation • Improvement of organoleptic properties
Bovine adipose-derived mesenchymal stem cells	Cultured meat marbling model [14]	• Formation of fat-rich edible tissue • Mimicry of inter- and intramuscular fat structures • Improvement of meat-like texture and quality
*Coffea arabica* cells	Cell culture-based coffee model [15]	• Generation of coffee-like aroma and flavor • Chemical and sensory resemblance to conventional coffee
*Sorbus aucuparia* and *Rubus arcticus* cells	Plant cell culture food model [16]	• Improvement of mouthfeel, texture, and flavor • Production of protein- and fiber-rich cell biomass

**Table 2:** Functional outcomes of bacterial SCPs.

Single cell type	Target model [Ref.]	Functional outcomes
*Methylococcus capsulatus*	Spotted sea bass [18]	• Alleviation of soybean-meal-induced enteritis • Modulation of intestinal immune responses • Modulation of gut microbiota
*Clostridium autoethanogenum*	Broilers [20, 21]	• Improvement of growth performance • Modulation of lipid metabolism and gut microbiota • Stimulation of muscle protein synthesis
*Corynebacterium glutamicum*	Pilot scale production [22]	• High protein content • DIAAS of 102
	Gilthead sea bream [23]	• Protein replacement without growth impairment • Maintenance of protein digestibility • Maintenance of intestinal morphology and gut microbiota
	Nursery pigs [24-26]	• Improvement of nutrient utilization • Modulation of jejunal immune and oxidative-stress status • Support of intestinal health and growth
	Small pet dogs [27]	• Modulation toward a healthier gut microbiota profile
	High-fat diet-induced obese mice [28, 29]	• Reshaping of gut microbiota • Modulation of adipose tissue distribution • Improvement of lipid- and gut-health-related parameters
	Porcine and bovine cell-culture models [30, 31]	• Support of heme tolerance • Adaptation to reduced-serum or serum-free culture • Induction of redox- and detoxification-related responses
	Caco-2 cells and DSS-induced colitis model [32]	• Repair of intestinal barrier dysfunction • Reduction of inflammatory and oxidative-stress responses • Activation of FOXO-associated repair pathways
*Akkermansia muciniphila*	Obese and diabetic mice [33]	• Improvement of obesity- and diabetes-related metabolic parameters • Enhancement of gut-barrier-related responses
	Overweight or obese insulin-resistant humans [34]	• Improvement of insulin sensitivity and insulinemia • Reduction of total cholesterol • Improvement of inflammatory and gut-barrier-related markers

**Table 3:** Functional outcomes of yeast and filamentous fungal SCPs.

Single cell type	Target model [Ref.]	Functional outcomes
*Cyberlindnera jadinii*	Zebrafish [35, 36]	• Modulation of intestinal immunoregulatory pathways • Improvement of immunity • Increased survival against *Aeromonas* infection
	Post-weaning piglets [38-40]	• Modulation of intestinal microbiota • Increase in butyrate-related responses • Support of post-weaning intestinal adaptation
	Labrador retrievers [41]	• Improvement of digestibility-related outcomes • Modulation of inflammatory markers • Reduction of fecal microbiota dysbiosis
*Saccharomyces cerevisiae*	Channel catfish [42]	• Improvement of growth performance • Enhancement of immunity and disease resistance • Modulation of intestinal microbiota composition and function
*Yarrowia lipolytica*	Pacific white shrimp [43] Largemouth bass [44]	• Support of growth at appropriate inclusion levels • Improvement of antioxidant capacity • Modulation of intestinal morphology and microbiota
	Nile tilapia [45] Rainbow trout [46]	• Stimulation of innate immune responses • Upregulation of intestinal immune-related genes • Gut-health-related modulation
*Debaryomyces hansenii*	Gilthead sea bream [47]	• Improvement of growth and feed conversion • Modulation of gut microbiota • Enhancement of host–microbiota interactions
*Fusarium venenatum*	Human [49, 50, 52-56]	• Promotion of postprandial amino acid availability • Stimulation of myofibrillar protein synthesis • Support of resistance-training adaptations
	Human [57-62]	• Reduction of cholesterol-related markers • Modulation of postprandial glucose responses • Improvement of gut fermentation and microbial profiles
*Paecilomyces variotii*	Atlantic salmon [63, 64]	• Maintenance of growth and feed intake • Improvement of feed conversion and nutrient retention • Modulation of gut health, immunity, and microbiota
	Adult dogs [65]	• Acceptable protein digestibility • Modulation of fecal short-chain fatty acids • Modulation of gut microbiota

**Table 4:** Functional outcomes of algal SCPs.

Single cell type	Target model [Ref.]	Functional outcomes
*Arthrospira platensis*	Pacific white shrimp [67]	• Improvement of growth-related traits • Enhancement of antioxidant activity and tissue condition • Increased resistance to *Vibrio parahaemolyticus*
	Zebrafish [69]	• Modulation of growth, reproduction, and metabolic responses
	Broilers [70, 71]	• Modulation of immune and antioxidant status • Gut microbiota-related effects • Protection against aflatoxin-associated pathological damage
*Chlorella vulgaris*	Aquatic animals [72-75]	• Modulation of antioxidant and immune responses • Improvement of intestinal function and gut microbiota • Enhancement of stress or disease resistance
	Weaned piglets [76]	• Reduction of diarrhea • Modulation of inflammatory and lipid-related parameters • Alteration of colonic microbiota and metabolites
*Nannochloropsis oceanica*	Atlantic salmon [77]	• Maintenance of EPA and DHA deposition • Partial replacement of conventional marine ingredients • Formulation-dependent effects on lipid digestibility
	Broilers [78]	• Enrichment of meat n-3 polyunsaturated fatty acids • Increased carotenoid deposition • Maintenance of meat-quality traits
	Rats [79]	• Increased lean mass • Reduced fat mass • Limited broader metabolic correction
*Nannochloropsis oculata*	Nile tilapia [80-82]	• Improvement of growth and feed utilization • Enhancement of antioxidant, digestive, and immune responses • Improvement of intestinal integrity and disease resistance
